# Preparation and Identification of Novel Antihypertensive Peptides from the In Vitro Gastrointestinal Digestion of Marine Cobia Skin Hydrolysates

**DOI:** 10.3390/nu11061351

**Published:** 2019-06-15

**Authors:** Yu-Hsin Lin, Chun-An Chen, Jenn-Shou Tsai, Guan-Wen Chen

**Affiliations:** 1 Department of Food Technology and Marketing, Taipei University of Marine Technology, No. 212, Section 9, Yan Ping North Road, Taipei 111, Taiwan; yhlin@mail.tumt.edu.tw; 2 Department of Food Science, National Taiwan Ocean University, No. 2 Pei-Ning Road, Keelung 202, Taiwan; anguschen823@gmail.com (C.-A.C.); tsaijs@mail.ntou.edu.tw (J.-S.T.)

**Keywords:** marine fish cobia, gastrointestinal digestion, inhibitory efficiency ratio, systolic blood pressure

## Abstract

This research focuses on cobia skin hydrolysates and their antihypertensive effects via the inhibitory activities of angiotensin I-converting enzyme (ACE). Marine fish Cobia (*Rachycentron canadum*) skin was hydrolysed for 5 h using Protamex and Protease N to obtain the cobia skin protein hydrolysates PX-5 and PN-5, respectively. The soluble protein and peptide contents of the PX-5 were 612 and 270 mg/g, respectively, and for the PN-5, 531 and 400 mg/g, respectively. The IC_50_ of PX-5 and PN-5 on ACE was 0.221 and 0.291 mg/mL, respectively. Increasing the IC_50_ from 0.221 to 0.044 mg/mL by simulated gastrointestinal digestion (PX-5G) reduced the ACE-inhibitory capacity of PX-5. Using gel filtration chromatography, the PX-5G was fractioned into eight fractions. The molecular weight of the fifth fraction from PX-5G was between 630 and 450 Da, and the highest inhibitory efficiency ratio on ACE was 1552.4%/mg/mL. We identified four peptide sequences: Trp-Ala-Ala, Ala-Trp-Trp, Ile-Trp-Trp, and Trp-Leu, with IC_50_ values for ACE of 118.50, 9.40, 0.51, and 26.80 μM, respectively. At a dose of 600 mg PX-5 powder/kg body weight, in spontaneously hypertensive rats PX-5’s antihypertensive effect significantly reduced systolic and diastolic blood pressure by 21.9 and 15.5 mm Hg, respectively, after 4 h of oral gavage.

## 1. Introduction

Globally, cardiovascular disease is a leading cause of death. Hypertension is a major risk factor for cardiovascular disease. The World Health Organization predicts that in 2020, stroke and heart disease will surpass infectious diseases as a cause of death [1]. One approach to addressing hypertension is the use of synthetic drugs to inhibit the activity of angiotensin I-converting enzyme (ACE). ACE is a key enzyme that regulate blood pressure in the human renin-angiotensin system. In medical operations ACE inhibitory drugs such as Capoten, Vasotec, and Aceon [2] are commonly used. These are largely short-chain peptide derivatives with verified effectiveness [3]. However, their side-effects may include dizziness, coughing, and allergic reactions [4]. Therefore, bioactive natural substances for inhibiting ACE functions are being explored.

ACE-inhibitory peptides have been the target of research in the last decade. These are produced via protease-hydrolysed food proteins. Researchers have obtained ACE inhibitory peptides from foods [5] such as fermented milk [6,7,8], shellfish [9,10], chicken [11], eggs [12,13,14,15,16], mushrooms [17], chlorella [18,19,20], fish [21,22], and fish scales and skin [23,24]. Gelatin extracted from the skin of squid has been hydrolysed by pepsin to produce ACE-inhibitory peptides. In the gelatin hydrolysates of the squid skin, a fraction with a desired low molecular weight (MW) peptide of less than 2 kDa revealed the strongest inhibitory capacity on ACE, with an IC_50_ value of 0.33 mg/mL. Oral gastric intubation was then used to administer the collected fraction to a rat model of renovascular hypertension (RHR). After 30 days, the fraction had a significant effect on blood-pressure reduction [25]. In another study, squid skin collagen was processed to yield the protein hydrolysates and then further hydrolysed by gastrointestinal proteases. Within the hydrolysate fractions, the strongest inhibitory capacity on ACE was exhibited by a low MW collected fraction (<1 kDa). After further isolation and sequence analysis of this collected fraction, its peptide sequence was revealed to be G–R-G-S–V-P–A–Hyp–G-P, which had an IC_50_ of 47.78 μM [26]. Gelatin extracted from Pacific cod (*Gadus microcephalus*) skin has been hydrolysed using gastrointestinal proteases, and commercial enzymes. Pacific cod skin gelatin hydrolysates (<1 kDa) by gastrointestinal proteases hydrolysis that produced two strong ACE inhibitory peptides were G-A-S-S-G-M-P-G and L-A-Y-A, which had IC_50_ values of 6.9 and 14.5 μM, respectively [27]. Cobia (*R. canadum*) head protein hydrolysate (CHPH) with an ACE-inhibitory effect has been prepared using papain. In the hydrolysate, a collected fraction with a low MW below <3 kDa (CHPH-IV) exhibited a reduced ACE IC_50_ value of 0.17 mg/mL (from 0.24 mg/mL) after hydrolysis in digestive enzymes. Spontaneously hypertensive rats (SHR) exhibited a significant dose-dependent reduction in blood pressure of after oral gastric intubation with CHPH-IV for 4 h at dose levels of 150, 600, and 1200 mg/kg body weight (BW) [28].

Cobia is a member of the Rachycentridae, a family of large predatory, pelagic oceanic fish. Except for the Eastern Pacific, it is primarily a denizen of tropical and subtropical waters [29], and is among the most commonly farmed fish in Penghu and Pingtung Counties, Taiwan [30]. In 2017, the production of cobia was 1615 metric tons, and its output value was NT$624.6 million [31]. Fresh cobia is usually served as sashimi or exported, but fillet production leaves numerous byproducts such as the head, liver, fin, and frame, roughly 40–50% of the fish by body weight [32]. In addition, more than 50% of byproducts of marine fish cobia are considered as waste after fishing and processing, so about 3.2 million tons of waste each year cause environmental pollution [33]. The fish skin and belly are used in salads and thus have relatively low economic value. The protein content of cobia skin is approximately 30%, making it an ideal candidate for developing functional foods that could add value to the cobia farming process.

Fish byproducts are highly nutritious and safe food ingredients [34] that can be used to produce foods and medicines, making them a crucial biological resource. Enzymes have been used to hydrolyse fish byproducts and prepare peptides with bioactivity [24,35,36]. Studies investigating the antioxidant and antihypertensive activities of compounds exhibiting bioactivity and peptides beneficial to health in fish skin and bone have attracted public interest [37,38,39]. However, little is known about the ACE-inhibitory activity and antihypertensive effects of protein hydrolysate from cobia skin. Because of the large quantities of proteins in cobia skin, bioactive peptides produced by protease hydrolysis of cobia skin are of great significances. Thus, to compare the efficacy of the hydrolysates, the current study measures the inhibitory effects of cobia skin protein waste on ACE. Simulated gastrointestinal digestion was used to measure the stability of the hydrolysates’ ACE-inhibitory effects. Furthermore, the major ACE inhibitory peptides of hydrolysates were purified and identified to confirm their peptide sequences. Short-term hypotensive effect of bioactive substance was then evaluated using SHRs as animal model.

## 2. Materials and Methods

### 2.1. Materials

*R. canadum* was purchased from Tan Hou Ocean Development Co. (Penghu, Taiwan), Taipei. Protamex (a mixture of endo and exopeptidase from *Bacillus* sp., activity labelled 1.5 AU/g) and Protease N (a mixture of endo and exopeptidase from *Bacillus subtilis*, activity labelled 150,000 U/g) were respectively obtained from Novo Nordisk A/S Co. (Bagsværd, Denmark) and Amano Pharmaceutical Co. (Yokohama, Kanagawa, Japan). Enzymes including pepsin, pancreatin and rabbit lung ACE were purchased from Sigma Chemical Co. (St. Louis, MO, USA). Substrate (hippuryl-L-histidyl-L-leucine, HHL) and all chemicals used were of analytical grade and also purchased from Sigma Chemical Co. (St. Louis, MO, USA).

### 2.2. Preparation of Hydrolysates of Cobia Skin

The cobia skin was immersed in boiling water (98 ± 2 °C) for 10 s and then cooled in cold water (4 °C) for 1 min, scales and remaining flesh were removed with a spoon. The skin was homogenised using a Blixer-3 (Robot Coupe Inc., (Ridgeland, MS, USA) at a high speed for 30 s, placed in a polyethylene bag, and stored at −20 °C. A total of 150 g of the blended fish skin was prepared and uniformly mixed with 6 times (*w*/*w*) deionized water containing with 1.35 g of commercial protease (Protamex or Protease N); the amount of protease added to the blended fish skin was 3% (*w*/*w*) of the fish-skin crude protein (30%). The mixture was left to hydrolyse at 50 °C in thermostatically water controlled water bath for 5 h. After hydrolysis, the mixture was immersed in a boiling water bath for 10 min to deactivate the protease and then cooled to room temperature in a cold water bath. The hydrolysate was filtered twice through even layers of diatomaceous earth placed evenly on Advantec No. 2 filter paper (Toyo Roshi Kaisha, Ltd., Tokyo, Japan). The clear translucent hydrolysate was freeze dried in Labconco freeze drying system (Labconco, AST Instruments Corporation, Kansas, KS, USA) to yield a powder for subsequent analysis.

### 2.3. Biochemical Analysis

The method developed by Folin–Lowry [40,41], was used to determine the content of soluble protein in the cobia fish skin hydrolysates, the standard used was albumin from bovine serum. One mL of copper analytical reagent (alkaline) was added as well as 3.0 mL of a 1:10 dilution of Folin–Ciocalteu reagent (Merck, KGaA, Darmstadt, Germany). Incubation was allowed to proceed for 30 min at room temperature. A Shimadzu UV spectrometer (Kyoto, Japan) was used to measure absorbance at 540 nm.

### 2.4. Determination of Peptides

A slightly modified procedure, developed by Church et al. [20,42], was used to measure the sample peptide content. A hydrolysate solution with a concentration of 30 mg/mL was passed through a 0.22-μm membrane before ultra-filtration at 5-kDa MW cut-off (MWCO). Fifty milliliter was mixed with 2.0 mL of *o*-phthaldialdehyde at room temperature and allowed to stand for 2 min. A Shimadzu UV-160A (Kyoto, Japan) was used to measure absorbance at 340 nm. The peptide content was quantified using Leu-Gly (Sigma, St. Louis, MO, USA) as a standard.

### 2.5. Measurement of Free Amino Acid Content

The free amino acid content of the sample was determined using a slight modifications of previously published methods [6,43]. A total of 0.5 mL of the protein hydrolysate was mixed with 1.0 mL of Cd–ninhydrin solution (0.8 g of ninhydrin dissolved in a mixture of 80 mL 99.5% ethanol and 10-mL acetic acid combined with 1.0 g of CdCl_2_ dissolved in 1.0 mL of deionized water). After addition of the sample the screw capped tube was rapidly transferred to a water bath at 84 °C for 5 min. After cooling to 25 °C, the absorbance was measured at 507 nm with a UV-160A spectrophotometer (Shimadzu, Kyoto, Japan). The free amino acid content was quantified using l-Leucine as a standard.

### 2.6. ACE Inhibition Assay

RP-HPLC was used to measure ACE inhibition by means of a method devised by Cushman and Cheung [44,45]. The sample consisted of 15 mM HHL in Na-borate buffer (100 mM, pH 8.3) plus NaCl (300 mM). Rabbit lung ACE, also in Na-borate buffer, was added to a concentration of 53.2 mU/mL. Equal volumes (75 µL) of the ACE solution and samples passed through a 5-kDa MWCO membrane were then incubated for 10 min at 37 °C. Seventy-five milliliter of the solution of HHL was then added and incubation was continued for a further 30 min. 1.0 N HCl (250 μL) was used to stop the reaction and 10 μL was injected into a Luna C_18_ column. The column used to separate the HHL substrate from the HA (hippuric acid) released by ACE action was a Phenomenex 5 µm (4.6 × 250 mm^2^). Elution of the column was done using 50:50 (*v*/*v*) methanol—0.1% trifluoroacetic acid at a constant flow rate of 0.8 mL/min using a Hitachi L-7100 pump (Tokyo, Japan) and monitored at 228 nm by a spectrophotometer using a UV-VIS 118 Gilson detector (Villiers-le-Bel, France). Finally, inhibition activity was calculated using the following formula:

Inhibition activity (%) = ((Ec − Es) / (Ec − Eb)) × 100

where Ec is the absorbance when the buffer was added as control, Es is the absorbance when the sample was added to the reaction mixture (sample), and Eb is the absorbance when the stop solution was added before the reaction occurred (blank). The IC_50_ value was defined as the peptide concentration (mg/mL) required to reduce 50% of the height of the HA peak (50% ACE inhibition) Evaluation of the IC_50_ was done using regression analysis of the ACE inhibition activity (%) against the log_10_ concentration (mg/mL) of the sample peptide. Captopril, used as a positive control, had significantly high ACE inhibition activity (IC_50_ = 0.0069 μM). The data used in this study were all averages of three readings or standard deviations (SDs).

### 2.7. In Vitro Gastrointestinal Digestion

In vitro digestion simulation was done using slight modifications of a previously published method [45]. The control used was 3.5% PX-5 (*w*/*v*) (hydrolysate from Protamex hydrolysis for 5 h) in 0.1 M KCl-HCl buffer (at pH 2.0) plus pepsin (1:25) incubation was carried out at 37 °C for four hours. The reaction was stopped by immersing the reaction vessel in boiling water for ten minutes. NaOH (2 M) was used to neutralize the mixture and 50 mL was spun at 10,000×*g*, for thirty minutes. The supernatant (2 mL) was analysed for ACE inhibition activity. The rest of the suspension was digested with pancreatin at 1:25 (*w*/*w*) for four hours at 37 °C. The vessel was immersed in boiling water for ten minutes to stop the reaction. The suspension was spun at 10,000×*g* for thirty minutes. Some of the supernatant was analysed for ACE inhibition and the rest was freeze dried to give PX-5G for later purification and analysis of ACE inhibition.

### 2.8. Gel Filtration

The hydrolysate of PX-5G had the highest ACE inhibition and was purified using Sephadex (G-25, 1.6 × 90 cm^2^; Amersham Pharmacia Biotech AB, Uppsala, Sweden) in a gel filtration process and 0.02% NaN_3_ in deionized water was used for equilibration. Three hundred milligram of hydrolysate in 10 mL of deionized water was filtered using a 5-kDa MWCO membrane and 2.0 mL of filtrate was injected into the Sephadex G-25 column (Amersham Pharmacia Biotech AB, Uppsala, Sweden) and eluted with 0.02% NaN_3_ at 0.5 mL/min. 5-mL fractions were collected, and the absorbance of each fraction was determined at 280 nm. The standards (Bacitracin, penta-L-phenylalanine and tryptophan) used for calibration of the Sephadex G-25 and their molecular weight were 1422 Da, 753.9 and 204.2 Da, respectively.

### 2.9. Isolation of Bioactive Peptides with ACE-Inhibitory Activity

A slight modification of the method developed by Chen et al. [6] was used to purify the peptides with ACE inhibitory activity from the hydrolysates. The fraction from the Sephadex gel filtration with the highest ACE-inhibitory activity was collected, lyophilized, and further separated by RP-HPLC (L-7100, Hitachi, (Tokyo, Japan)) using a semi-preparative C_18_ column (Synergi 4μm Hydro-RP 80 Å, 10 × 250 mm^2^; Phenomenex, (Torrance, CA, USA)). Solution A and solvent B were 0.1% trifluoroacetic acid (TFA) in deionized water and acetonitrile solution containing 0.1% TFA. A gradient from 0% to 50% of solvent B was employed, with a sample of 500 µL, to separate the peaks using a flow of 1.5 mL/min over two hours at room temperature. A Gilson 118 UV–VIS detector and a 715 system controller were used and monitoring was done at 220 nm. Repeated runs were done to collected the individual peaks. Each was tested for purity in the same way as described above using a 4 µm Joupiter Proteo (Phenomenex, Torrance, CA, USA) 90Å, column, the same solvent, gradient and flow rate over one hour. Monitoring was done during elution at 220 nm. Finally, the peaks were collected and lyophilized to a powder for ACE inhibitory activity assay, and then the peaks demonstrating the highest inhibition of ACE were collected and lyophilized, and their peptide sequences were identified.

### 2.10. Identification of Antihypertensive Peptide Sequences

A slight modification of the method offered by Lin et al. [20,46] was used to determine the peptide sequences. Samples for analysis were prepared as follows: The PX-5G concentration was increased from 30 to 100 mg/mL and purified by gel filtration chromatography. Fraction E was collected through triplicate chromatography. The collections were combined, lyophilized, and dissolved in 0.5 mL of deionized water. The resulting solution was further separated by RP-HPLC on a semi-preparative C_18_ column using the method described before. Each peak was collected using quintuplicate chromatography, and then a single component of each peak was confirmed using an analytical C_12_ analytical column by the previously described method. The five collected mixtures were then lyophilized, and their inhibition of ACE activity and peptide sequence was determined. Automated Edman degradation was done using a Procise 492 protein sequencer (Perkin-Elmer Co. Ltd., Applied Biosystem Inc., Foster City, CA, USA) to determine the sequence of amino acids in peptides that showed high ACE inhibition [40]. Finally, the identified ACE-inhibitory peptides were synthesised through solid-phase peptide synthesis. The synthetic peptides were used as the standard for qualitative analysis of these peptides in the cobia skin hydrolysates using an RP-HPLC column (ODS C_12_) by the previously described method. Amino acid sequence alignment of the *R. canadum* proteins (Secreted protein acidic and cysteine rich, SPARC (accession number: G8XR48), rhodopsin (accession number: T1QSV8), elongation of very long chain fatty acids protein 5 (accession number: B7U6V2), and cytochrome c oxidase subunit 1 (accession number: B5U133)) was done using the UniProt database [47]. Pairwise sequence alignment tools were employed to confirm identical sequences [48].

### 2.11. Blood Pressure Measurement

The scientific research and testing using animals were conducted according to the rules and regulations of the National Taiwan Ocean University, Animal Care and Use Committee, Keelung, under ethical approval certificate number 95,028. Eighteen seven weeks old male SHRs were kept in individual steel cages with a 12-h light and dark cycle. The ambient temperature and relative humidity were 23 ± 1 °C and 55% ± 5%. The feed used was Rodent Laboratory 5001 from PMI Nutrition International, Brentwood, MO, USA. The animals had free access to water and were kept for eight weeks before the experiments were started. The fifteen week old SHRs had a body weight of 350g ± 5 g, systolic blood pressure of 173.0 ± 4.2 mm Hg and diastolic blood pressure of 150.0 ± 3.7 mm Hg. In animal studies, the blood pressure was obtained from eighteen rats. The extreme values, including the four highest and four lowest, were excluded. The remaining values were randomly allocated into the control group (*n* = 5) and study group (*n* = 5). A statistical analysis showed no significant difference in the systolic and diastolic pressures of the two groups (*p* < 0.05). This was followed by a short-term rat gavage study to assess the blood pressure changes. Two groups of five rats were selected at random and one group received gastric intubation of PX-5 dissolved in 2 mL of saline at a dose rate of 210 mg per 350 g BW (56.7 mg peptide/rat BW). The second group of five received equal volumes of normal saline. The systolic blood pressure (SBP) and diastolic blood pressure (DBP) of the animals was measured at two hourly intervals for eight hours and then at 24 h after dosage. Each animal was placed in a box controlled at 45 °C for 5 min before blood pressure and heart rate measurements were made. A noninvasive method, BP-98, Softron, Tokyo, Japan, was used with a tail-cuff. The results are shown as means ± SDs.

### 2.12. Statistical Data Treatment

Changes in blood pressure were determined as the difference between systole and diastole before/after dosage with 56.7 mg of peptide/rat BW of PX-5 (data expressed as mean ± SD). The analytical results of the cobia skin hydrolysates obtained by gel column size exclusion and RP-HPLC are averages of the measurement of 3 samples. All statistical analyses were carried out using SAS (version 9.4 TS1M5; SAS Institute Inc., Carry, NC, USA). The *p*-value less than 0.05 was considered statistically significant. SAS was used for the analysis of variance calculations using the general linear procedure [49]. SAS was also used for Duncan’s multiple range multiple mean test comparisons. 

## 3. Results and Discussion

### 3.1. IC_50_ and Soluble Protein, Peptide, and Free Amino Acid Contents

The scales and residual flesh on a cobia skin sample were removed and it was hydrolysed using protease, specifically Protamex and Protease N, for 5 h to give hydrolysates PX-5 and PN-5. The chemical compositions (soluble protein, peptide, and free amino acid contents) as well as ACE IC50 of the hydrolysates were subsequently determined (Table 1). In this study cobia skin was hydrolysed for 2 h (data not shown) and 5 h with Protamex and Protease N and then determined their contents of soluble proteins, peptides and free amines. In the 2-h and 5-h hydrosylate with Protamex, the levels of soluble proteins are 555.0 ± 4.0 and 612.0 ± 9.0 mg/g, respectively, and the levels of peptides are 180.0 ± 3.0 and 270.0 ± 7.0 mg/g, respectively. For the Protease N group, the levels of soluble proteins are 498.0 ± 5.0 and 531.0 ± 7.0 mg/g, respectively, and the levels of peptides are 258 ± 6.0 and 400.0 ± 9.0 mg/g, respectively. When hydrolysed by these proteases, the level of each composition increases with time. This result is similar to the changes of composition level seen in the hydrosylates obtained by treating clam flesh with Protamex [50]. With longer treatment time, larger protein molecules are hydrolysed, thus the levels of soluble proteins and peptides increase. In addition, the results showed that protein hydrolysate PX-5 (612 mg/g) contained a higher soluble protein content than did PN-5 (531 mg/g). By contrast, PN-5 had higher peptide and free amino acid contents than did PX-5, with the peptide content of PN-5 (400 mg/g) being 1.5 times that of PX-5 (270 mg/g). The ACE-inhibitory activity of PX-5 (IC_50_ = 0.221 mg/mL) was higher than that of PN-5 (IC_50_ = 0.291 mg/mL).

### 3.2. Antihypertensive Effect of Cobia Skin Protein Hydrolysate

**Figure 1:**
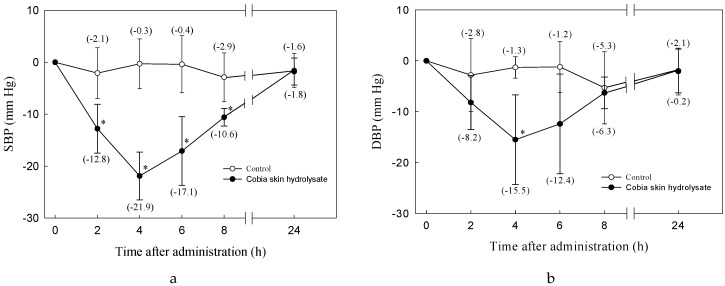
The effect of oral administration of PX-5 on blood pressure in SHRs. (**a**) systolic blood pressure (SBP); (**b**) diastolic blood pressure (DBP). —○—, control = 0.9% NaCl in deionized water; —●—, 210 mg of hydrolysate in 0.9% NaCl; Each value is a mean of five readings. The bars indicate standard error. *: significant difference from control, *p* < 0.05.

PX-5 (56.7 mg of peptide/350 g BW) was given to SHRs over a short term. This was the equivalent of 350 g—equivalent to 600 mg powder/kg BW/rat. Because of its negligible effect on SBP, saline was used as a control. PX-5 administration caused a significant decrease in SBP after 2 to 8 h which reverted to the original level after 24 h. SBP in the SHRs that had received the peptide was 21.9 mm Hg lower than that of the controls 4 h after feeding. For DBP the values in both experimental groups was 15.5 mm Hg lower than in the controls, see Figure 1.

Other reports about cobia protein hydrolysates [28], such as CHPH-IV extracted from cobia head protein hydrolysate by papain, showed strong antihypertensive action in SHRs. At dosages of 150, 600, and 1200 mg of hydrolysate/kg BW, SBP decreases of about 30, 35 and 57 mm Hg were observed four hours after dosing. Tofuyo also showed strong antihypertensive action. SHRs fed a diet containing lyophilized tofuyo for 6 weeks, showed an SBP decrease of 8 mm Hg. The major ACE-inhibitory peptide separated from tofuyo is Trp-Leu [51]. Other studies have revealed the experimental results of feeding unique tryptophan-containing peptides to humans. Baseline plasma concentrations of the Trp-Leu peptide measured after oral administration of 100 mg Trp-Leu to human volunteers were reported in detail by Kaiser et al. [52]. The maximum plasma concentrations of Trp-Leu were approximately 29–36 pmol/mL after 30 min, with ACE activity in plasma decreasing by 16–22% at 1.5 h [52]. These results showed Trp-Leu is resistant to enzymatic hydrolysis in the stomach and intestines and can enter the blood circulatory system. These results can serve as a reference for our future research.

### 3.3. In Vitro Digestive Stability and ACE inhibition of the Cobia Skin Hydrolysates

Simulated gastric digestion in vitro was considered a useful and simple method to determine the degree of resistance of these ACE-inhibitory peptides to degradation. The ACE-inhibitory activity of both PX-5 and PN-5 were decreased significantly by simulated gastrointestinal (pepsin–pancreatin) hydrolysis. The IC_50_ values increased from 0.221 to 0.304 mg peptide/mL and that of PN-5 from 0.291 to 0.384 mg peptide/mL (see Table 2). While hydrosylates PX-5 and PN-5 showed reduced ACE inhibition from the hydrolytic activity of digestive enzymes, PX-5 still showed better ACE inhibition than PN-5. Therefore, PX-5 was chosen for the peptide purification and identification analysis. In another study the ACE inhibition effects of *Chlorella sorokiniana* peptides went down after simulated gastrointestinal digestion, and the IC_50_ rose from 0.035 to 0.044 mg peptide/mL [20]. In addition, the major peptide inhibitor of ACE produced from Manchego cheese through separation and identification was sequenced as Val-Arg-Tyr-Leu, corresponding to the α_S2_-casein fragment f (205–208). This peptide was further hydrolysed with gastrointestinal proteases, after which its IC_50_ value raised from 0.009 to 0.03 mg/mL. However, the ACE-inhibitory capacity decreased because the hydrophobic amino acid leucine at the C-terminus of Val-Arg-Tyr-Leu underwent hydrolysis and generated Val-Arg-Tyr [53].

### 3.4. Separation and Assessment of Potent ACE-Inhibitory Peptides

**Figure 2:**
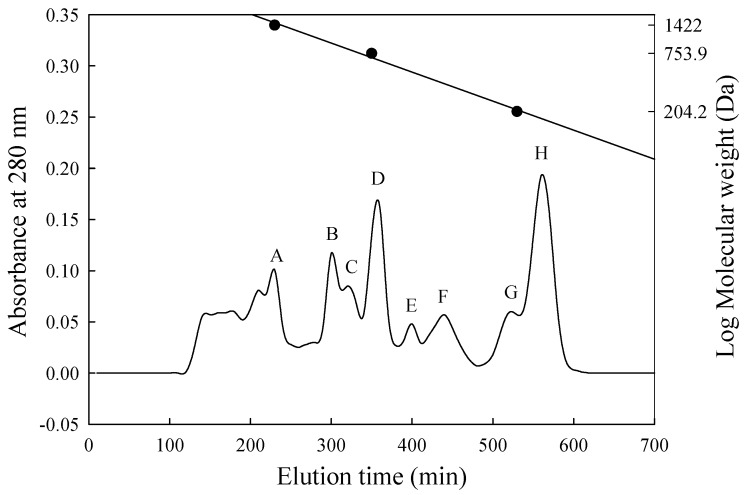
Peptides separate from PX-5G on a Sephadex G-25 column. ● Standards: The MWs of Bacitracin, penta-L-phenylalanine and L-tryptophan were 1422, 753.9 and 204.2 Da, respectively. PX-5G was separated into 8 fractions designated as A-H.

In this study, the ACE-inhibitory capacity of PX-5 decreased (the IC_50_ value rose from 0.221 to 0.304 mg/mL) after enzymatic hydrolysis in the stomach and intestines. This result suggests that proteases present in the stomach and intestine can hydrolyse the peptide sequences of ACE-inhibitory activity in PX-5 mixtures. PX-5 after digestion by gastrointestinal proteases (PX-5G) was selected for the purification and identification of possible ACE-inhibitory peptides obtained from the in vitro model simulating gastric digestion, we selected PX-5G to conduct purification and identification. The MW distribution of the ACE-inhibitory peptides in PX-5G was separated by size exclusion chromatography on a Sephadex G-25 column. Eight fractions were separated with MWs ranging from 1670 to 160 Da, and were designated A–H (see Figure 2). 

The concentration of peptide in fractions A–H were 0.706, 1.190, 0.820, 0.397, 0.042, 0.028, 0.083, and 0.022 mg/mL, respectively (Table 3). In spite of fraction A had the strongest ACE-inhibitory percentage, the data for effective ACE inhibition (inhibitory efficiency ratio (IER) = inhibition (%)/peptide concentration (mg/mL)) indicated that the peptide fraction E showed a higher rate of inhibition than any from the other peaks. Specifically, the IER was 1553% per mg/mL (Table 3), and its IC_50_ value for ACE was 0.020 mg/mL (data not shown). The highest ACE-inhibitory activity was similar to that of the potent inhibitory tripeptides from the skin of *Theragra chalcogramma*, *Salmo salar* or *Oncorhynchus keta* [24,54,55]. According to previous research, many bioactive peptides with ACE-inhibitory activities are short peptide sequences and consisted of 2–12 amino acid residues [56].

**Figure 3:**
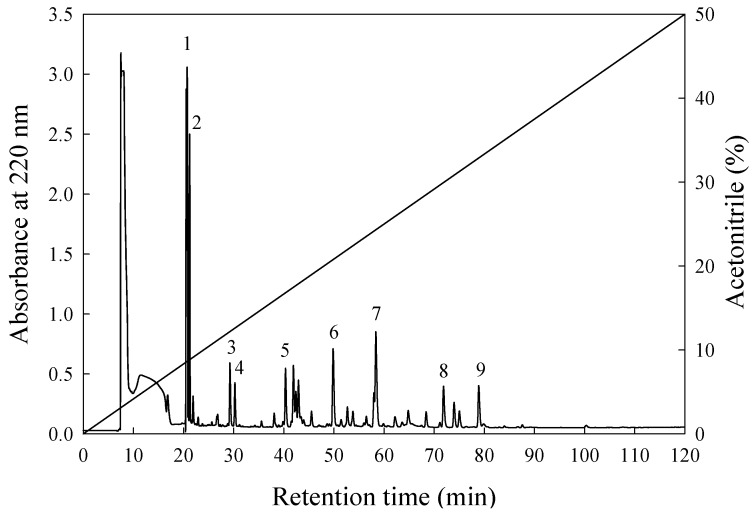
An elution profile analysis of fraction E from PX-5G by RP-HPLC.

In the present study, the most active peptide of fraction E was further purified on an RP-HPLC column (ODS C_18_). Figure 3 shows the elution profiles of the peptides. Nine major individual substances were collected using repetitive runs using an RP-HPLC C_12_ column and the same elution gradient as before. Each individual peak was found to be from a single component. Of the nine bioactive substances that had been found, it was determined from the IERs that bioactive substances E_6_, E_7_, E_8_, and E_9_ showed strong ACE inhibition activity with IERs of: 872.9%, 1398.3%, 3335.7%, and 3021.4% per mg/mL, respectively, see Table 4. These four peaks were collected, lyophilized, and their peptide sequences were determined.

### 3.5. Amino Acid Sequences and ACE-Inhibitory Activity

The amino acid sequences and IC_50_ values for the peptides from peaks E_6_, E_7_, E_8_, and E_9_ are shown in Table 5. The peptide sequences were: E_6—_Trp-Ala-Ala, E_7—_Ala-Trp-Trp, E_8—_Ile-Trp-Trp and E_9—_Trp-Leu. The IC_50_ values were: E_6—_118.50, E_7—_9.40, E_8—_0.51 and E_9—_26.80 μM this was equivalent to 0.0411, 0.00434, 0.00026, and 0.00851 mg/mL, respectively. These isolates were found to belong to the amino acid sequence of SPARC, rhodopsin, elongation of very long chain fatty acids protein 5, and cytochrome c oxidase subunit 1, residues 283–285, 226–228, 153–155, and 131–132, respectively [47]. Sekiya et al. [57] found that food peptides with ACE inhibition activity that had IC_50_ value from 100 to 500 μM had antihypertensive potential. Overall, it was shown that the ACE inhibition of five of the fractions (E, E_6_, E_7_, E_8_, and E_9_) from PX-5G could be raised by purification, either by size exclusion, or RP-HPLC. Cobia skin protein hydrolysates (0.304 mg/mL) that had not been purified had much higher IC_50_ values: Fraction E, Trp-Ala-Ala, Ala-Trp-Trp, Ile-Trp-Trp, and Trp-Leu, were respectively about 15, 7, 70, 1169, and 35 times lower.

The present study is the first to report the novel ACE-inhibitory peptide Trp-Ala-Ala. To the best of our knowledge, Ala-Trp-Trp and Trp-Leu are the only potent ACE inhibitors that have been identified to occur in a number of protein hydrolysates, for example from hydrolysates of soybean, tofuyo (fermented soybean curd), and salmon. These had IC_50_ values of 6.5 [54] and 29.9–34.1 μM [58,59], very close to the IC_50_ value of the purified peptide from PX-5G. However, the Ile-Trp-Trp prepared in this study had a lower the IC_50_ value than was obtained by Panyayai et al. (489.14 μM) [60]. This difference in IC_50_ values might be because authors used a spectrophotometric method [44,45,60] and a different substrate (furanacryloyl-Lphenylalanylglycylglycine, FAPGG) [61] for their ACE assays. Although other protein hydrolysates have yielded these peptides, no reports have revealed their presence in cobia skin proteins [62,63,64].

In this study the IC_50_ values of purified Trp-Ala-Ala and Trp-Leu were higher than those from other peptide sequences. However, a comparison of the IC_50_ values of Trp-Leu and its reverse sequence showed that the Trp-containing di-peptide at N-terminal residue exhibited lower ACE inhibition. Lower than that of Trp-containing di-peptides at C-terminal-residue with the IC_50_ values rising from 1.11 to 118.50 μM, as seen earlier [15]. This is consistent with the findings of Ono et al., with respect to dipeptides and the importance of amino acids at their carboxy terminal [59]. They identified Met-Trp and Trp-Met, different sequences composed of the same amino acid residues, from salmon muscle hydrolysate. The inhibitory activity of Met-Trp (IC_50_ = 9.8 μM) was 10 times greater than that of Trp-Met (IC_50_ = 98.6 μM). In some studies, it has been reported that the ACE-inhibition modes of di-peptide pairs (such as, VW, IW, and MW) containing the same amino acid compositions but reversed peptide sequences, were of different types, except for Trp-Leu and Leu-Trp, which were both noncompetitive inhibitors [59]. Some results in the literature indicated that the proper sequence and composition of amino acid residues are crucial in determining the ACE-inhibition mode of the peptides [59]. The Ala-Trp-Trp and Ile-Trp-Trp sequences also showed excellent ACE inhibition. This may have been because the C-terminals all included Trp-containing amino acids. The N-terminal on the other hand were all hydrophobic branched-chain amino acids. This result was similar to that found by He et al. [65]. Wu et al. [66] used Z descriptors in a study of ACE 168 dipeptides and 140 tripeptides with respect to quantitative structure/activity relationship. They found that ACE inhibition was influenced to a large extent by three-dimensional chemical properties. Hydrophobic peptides with terminal carboxy amino acids, like tyrosine, tryptophan, and phenylalanine are all know to have very good ACE inhibition. The peptide sequences all agreed with results obtained by Li et al. [67] for systematic induction and those of Cheung et al. [68] with respect to ACE inhibition by peptides. Panyayai et al. [51] also performed molecular docking to elucidate the catalytic pockets of ACE. The results showed that Ile-Trp-Trp could bind to the catalytic pockets of ACE through a network of hydrophobic, electrostatic, and hydrogen bonds with different carboxylic groups of ACE residues. This network could increase its interaction with ACE in a favorable way. Notably, the most advantageous type of each amino acid position in a tripeptide is unique. The preferred residue at the carboxy terminus is an aromatic amino acid, while the intermediate position is a positively charged amino acid, and the amino terminus is preferably a hydrophobic amino acid [66]. However, elucidation of the correlation between the mechanisms of ACE inhibition and the peptide structures await further study.

## 4. Conclusions

Cobia skin was hydrolyzed in Protamex for five hours. The IC_50_ of the resulting hydrolysate (PX-5) to ACE was 0.221 mg/mL. SHRs were given oral doses of 600mg of PX-5/kg BW. Four hours after administration, the systolic blood pressure of the experimental SHR group had dropped 21.9 mm Hg below than that of the controls and the diastolic blood pressure had dropped to 15.5 mm Hg lower than that of the controls. Four peptides from the hydrolysate that showed active ACE inhibition had amino acid sequences of: Trp-Ala-Ala, Ala-Trp-Trp, Ile-Trp-Trp, and Trp-Leu, and IC_50_ values of 118.5, 9.4, 0.51, and 26.8 μM. The ACE inhibition was present in vitro and clear antihypertensive activity was demonstrated in vivo. This suggested that an ACE-inhibitor could be derived from a cobia skin protein hydrolysate and used to develop functional products that could be used for the prevention of hypertension. This study also provides evidence that the small cobia skin peptides are bioactive and have potential for such applications.

## Figures and Tables

**Table 1:** Chemical compositions and ACE IC_50_ of cobia skin protein hydrolysates.

Sample	Soluble Protein	Peptide Content	Free Amino Acid (mg/g)	IC_50_^1^ (mg/mL)
	(mg/g)	(mg/g)		
PX-5 ^2^	612.0 ± 9.0 ^b^	270.0 ± 7.0 ^a^	75.5 ± 0.2 ^a^	0.221 ± 0.005 ^a^
PN-5 ^3^	531.0 ± 7.0 ^a^	400.0 ± 9.0 ^b^	97.3 ± 0.8 ^b^	0.291 ± 0.005 ^b^

Values are averages of three measurements. Superscripts in the columns signify notable differences between values. ^1^ Amount that will inhibit ACE activity by 50%. ^2^ PX-5 was produced from Protamex (3%) hydrolysis for 5 h. ^3^ PN-5 from five hours protease N (3%) hydrolysis.

**Table 2:** ACE activity inhibition of PX-5 and PN-5 obtained from gastrointestinal protease hydrolysis.

Sample	Protease	IC_50_ (mg/mL)
PX-5	Control	0.221 ± 0.005 ^b^
	PX-5P ^1^	0.217 ± 0.005 ^b^
	PX-5G ^2^	0.304 ± 0.005 ^a^
PN-5	Control	0.291 ± 0.005 ^c^
	PN-5P ^1^	0.308 ± 0.003 ^b^
	PN-5G ^2^	0.384 ± 0.002 ^a^

Values are averages of three measurements. Superscripts in the columns signify notable differences between values. ^1^ Four hours pepsin hydrolysis. ^2^ Four hours pancreatin hydrolysis after four hours of pepsin hydrolysis.

**Table 3:** ACE IC_50_ values of fractions obtained by size exclusion chromatography from PX-5 after gastrointestinal digestion.

Fraction	Molecular Weight (Da)	Inhibition (%)	Peptide Content (mg/mL)	IER ^1^ (%/mg/mL)
A	1670–1370	72.3	0.706	102.4
B	1130–870	56.5	1.190	47.5
C	870–760	71.8	0.820	87.6
D	760–630	57.2	0.397	144.1
E	630–450	65.2	0.042	1552.4
F	450–350	34.0	0.028	1214.3
G	270–220	22.2	0.083	267.5
H	210–160	22.2	0.022	1009.1

Values are averages of three measurements. ^1^ IER inhibitory efficiency ratio.

**Table 4:** Isolation of bioactive substances from Figure 2 and their ACE IER analysis.

Bioactive Substances	Inhibition (%)	Peptide Concentration (mg/mL)	IER^1^ (%/mg/mL)
E_1_	2.7	0.009	300.0
E_2_	6.5	─^2^	─^2^
E_3_	55.6	0.073	761.6
E_4_	15.3	0.109	140.4
E_5_	7.9	0.024	329.2
E_6_	41.9	0.048	872.9
E_7_	81.1	0.058	1398.3
E_8_	93.4	0.028	3335.7
E_9_	42.3	0.014	3021.4

Values are averages of three measurements. ^1^ IER inhibitory efficiency ratio. ^2^ Undetected.

**Table 5:** Identification of peptide sequences from E6 to E9 peaks and their ACE IC_50_ analysis.

Peak	Sequence	IC_50_ (μM)	IC_50_ (mg/mL)
E_6_	Trp-Ala-Ala	118.50 ± 2.80	0.04110
E_7_	Ala-Trp-Trp	9.40 ± 0.60	0.00434
E_8_	Ile-Trp-Trp	0.51 ± 0.10	0.00026
E_9_	Trp-Leu	26.80 ± 0.90	0.00851

Values are averages of three measurements.

## Abbreviations

PN-5	hydrolysate from Protease N hydrolysis for 5 h
PX-5	hydrolysate from Protamex hydrolysis for 5 h
ACE	angiotensin I-converting enzyme
MW	molecular weight
SBP	systolic blood pressure
DBP	diastolic blood pressure
SHRs	spontaneously hypertensive rats
BW	body weight
RP-HPLC	reverse-phase high-performance liquid chromatography
IER	inhibitory efficiency ratio
HHL	hippuryl- L-histidyl-L-leucine
MWCO	molecular weight cut-off
HA	hippuric acid
